# Association between Prenatal Adversity and Neonatal Outcomes in Infants with Prenatal Opioid Exposure

**DOI:** 10.64898/2026.10.08.26365058

**Published:** 2026-10-11

**Authors:** Madison Ramirez Heil, Macie Donahue, Marie Camerota, Lynne Dansereau, Alexandrea Craft, Elisabeth Conradt, Barry Lester

## Abstract

**Objective:**

Examine whether prenatal adversity is associated with Neonatal Opioid Withdrawal Syndrome (NOWS) severity and the need for pharmacological treatment in infants with prenatal opioid exposure.

**Study Design:**

Prospective cohort study of 235 infants from a multi-center study of infants with prenatal opioid exposure. Maternal factors were assessed using the electronic medical record and maternal questionnaires. Latent Class Analysis (LCA) was used to derive mutually exclusive prenatal adversity groups.

**Results:**

A 3-class LCA solution provided the best fit to the data and showed 3 distinct groups: one low adversity group (Class 3, 38.7%) and 2 high adversity groups. Infants born to mothers with elevated demographic, medical, polysubstance, and mental health risk factors (Class 1, 31.9%) were more likely to require pharmacological treatment for NOWS (OR=7.70, 95% CI: 3.65-17.14) and had higher NOWS severity, as indexed by longer length of stay (β = 6.08, 95% CI: 4.09-8.07), and higher maximum Finnegan scores (β = 2.62, 95% CI: 1.49-3.76). Infants born to mothers with elevated medical and polysubstance risk factors (Class 2, 29.4%) were also more likely to receive pharmacological treatment for NOWS (OR=3.22, 95% CI: 1.56-6.78) and had higher NOWS severity, as indexed by higher likelihood of receiving adjunctive medication (OR=3.62, 95% CI: 1.11-12.99).

**Conclusion:**

Maternal sociodemographic, mental health, substance use, and medical adversity may serve as an early marker of NOWS severity and may aid in the prediction of which neonates will require pharmacological treatment for NOWS. Early identification of infants at high risk for NOWS could lead to earlier treatment and the potential to reduce NOWS severity, length of stay, and associated healthcare costs.

## Full Text Availability

The license terms selected by the author(s) for this preprint version do not permit archiving in PMC. The full text is available from the preprint server.

